# The Need for More Mentorship in Medical School

**DOI:** 10.7759/cureus.7984

**Published:** 2020-05-06

**Authors:** Vikrant Bhatnagar, Sebastian Diaz, Philip A Bucur

**Affiliations:** 1 Family Medicine, Ohio University Heritage College of Osteopathic Medicine, Athens, USA; 2 Family Medicine, Ohio University Heritage College of Osteopathic Medicine, Dublin, USA; 3 Medicine, Ohio University Heritage College of Osteopathic Medicine, Athens, USA

**Keywords:** medical school, mentorship, gender differences, primary care, specialty medicine

## Abstract

Introduction

Mentorship, a supportive relationship that actively provides knowledge and insight, has many benefits. Although not extensively studied, medical students pursuing various specialties have diverse experiences with mentorship.

Objective

To understand how mentorship impacts medical student decisions involving rotation choices, residency programs, field of practice, interest in research, and career trajectory.

Methods

We hypothesized that effective mentor-mentee relationships would strongly impact medical students' decisions. Distributed to fourth-year osteopathic medical students at a single medical school, this study used a survey design to assess mentorship’s impact on their aforementioned decisions.

Results

Sixty-one students responded to this survey. Fifty-nine percent of respondents said they did not receive enough mentorship in medical school while 63.9% of respondents said their quality of mentorship was good/very good. Most survey respondents strongly agreed/agreed that the amount and quality of mentorship impacted their decisions involving rotation choices, residency programs, field of practice, and career trajectory. Qualitative data analysis led to the emergence of three themes: students pursuing primary care had positive mentorship experiences as compared to students pursuing non-primary care careers, female students stated they did not receive enough mentorship, and a majority of students cited the lack of formal mentorship as an area of improvement.

Conclusions

Our study demonstrates the profound impact mentorship has on a medical student’s career. Respondents believed their mentorship experiences strongly impacted their decisions involving rotation choices, residency programs, field of practice, and career trajectory. Participants also believed availability in the amount and quality of mentorship could be improved. The perceived lack in the amount and quality of mentorship may have negative implications on medical students’ career prospects.

## Introduction

Mentorship promotes career success in numerous ways [1]. In medicine, it impacts career selection, improves job satisfaction and compensation, and optimizes research productivity [1-3]. Physicians with mentors are twice as likely to be promoted as compared to physicians without mentors [1-3]. Mentoring medical trainees, such as resident physicians and medical students, increases research productivity, improves well-being, and develops an interest in a certain specialty [4]. Additionally, medical trainees with mentors are “twice as likely to state that they received excellent career preparation” [5].

Mentorship is an insightful process in which wisdom is acquired from the mentor in a supportive, protective relationship that necessitates active participation from both the mentor and the mentee [4]. It can develop through formal and informal mechanisms. Formalized mentorship is defined by the formal pairing of mentors and mentees from a third party [2]. Informal mentorship occurs through an organic interpersonal relationship between a mentor and mentee [2]. Either mechanism of mentorship can be effective, depending on the mentor and mentee.

The experience of mentorship by medical students has not been extensively studied. One survey of medical students at the University of California, San Francisco, found that only 36% of medical students had mentors [6]. Another study seeking to better understand why medical students pick certain medical specialties found that students inclined to pursue primary care were more likely to state that mentors positively and directly impacted their desire and inclination [7]. Medical students pursuing any specialty, primary care or non-primary care, felt that individuals who refute negative stereotypes of physicians in their desired field are a great influence in their decision of specialty choice [7].

Research reveals gender differences in medical mentorship. One study found that 40% of male medical students had mentors as compared to 33% of female medical students [6]. After medical students complete training, these gender differences in mentorship persist. Junior faculty clinicians (91% men versus 78% women) and staff physicians (84% of men versus 79% of women) experience differences in mentorship rates [8]. Additionally, male physicians are three times more likely than female physicians to describe their mentorship experience as having a positive impact on their career [8].

Given the importance of mentorship for success in medical professions, this study explores how mentorship impacts a medical student’s decisions involving rotation choices, residency programs, field of practice, interest in research, and career trajectory. Additionally, by focusing specifically on fourth-year medical students, these subjects can better reflect upon their mentor-mentee relationships in medical school and inform how and to what degree mentorship or its lack thereof affected their decisions.

We hypothesized, therefore, that students with constructive mentor-mentee relationships and experiences would reveal positive impacts on their decisions such as rotation choices, residency programs, field of practice, interest in research, and career trajectory. We also hypothesized that when asked to reflect on their mentor-mentee relationships, students having positive mentoring experiences would identify positive impacts on career decision-making.

## Materials and methods

This study utilized an anonymous, online, cross-sectional survey administered to fourth-year osteopathic medical students at a midwestern medical school. Institutional review board (IRB) approval was obtained to investigate mentorship’s impact on fourth-year osteopathic medical student decisions involving rotation choices, residency programs, field of practice, interest in research, and career trajectory. Respondents included fourth-year medical students from the 2017 and 2018 graduating classes. The anonymous online survey was administered using Qualtrics software (Qualtrics Inc, Provo, Utah). The survey was available for two weeks to allow respondents time to complete it. Participant anonymity was ensured through software settings preventing the collection of Internet protocol addresses.

The online survey (provided in the Appendix) consisted of nine questions, of which the first three questions focused on gaining consent and demographic information. Other questions addressed: 1) whether respondents had a relative who was a physician; 2) how much mentorship the participant received in medical school; 3) the perceived quality of mentorship the participant received in medical school; 4) whether the amount of mentorship they received impacted their decisions for rotation choices, residency programs, field of practice, interest in research, and career trajectory; 5) how the quality of mentorship they received impacted their decisions for rotation choices, residency programs, field of practice, interest in research, and career trajectory. The ninth question utilized an open-ended, narrative format question that asked, “Regardless of amount or quality of mentorship you received, briefly explain below how mentorship in medical school impacted your future career.” Data were analyzed using IBM Statistical Package for the Social Sciences (SPSS) statistical software version 24.0 (IBM Corp., Armonk, NY) and NVIVO version 12 PRO (QSR International, Doncaster, Australia).

## Results

Of the 265 (n=265) osteopathic medical students from a single osteopathic medical school who received the survey link over the two years, 61 (n=61) students responded, yielding a 23.0% response rate. Female students represented 52.5% of participants (Table 1). Participants in this survey were predominantly white (86.9%; n=53), while black/African American and Asian students combined represented 8.2% (n=5) of the sample (Table 1). Approximately 36.1% of participants had a relative who was a physician (Table 1).

**Table 1:** Demographics of the survey respondents with gender, race/ethnicity, and if a respondent had a physician as a relative

	n =	% of Respondents
Gender		
Female	32	52.5
Male	29	47.5
Total	61	
Race/Ethnicity		
White	53	86.9
Black/African American	3	4.9
Asian	2	3.3
Other	3	4.9
Total	61	
Physician as a Relative		
No	39	63.9
Yes	22	36.1
Total	61	

**Figure 1:**
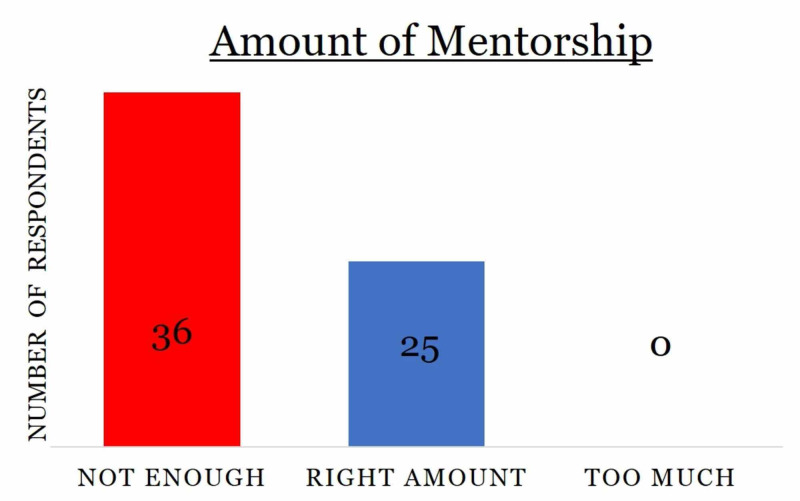
Distribution of survey respondents based on the amount of mentorship they received with the number of respondents listed within the chart

Fifty-nine percent of respondents (n=36) said they did not receive enough mentorship in medical school while the rest of the participants (41%; n= 25) said they received the right amount of mentorship (Figure 1). No respondent claimed to receive excessive mentorship (Figure 1).

**Figure 2:**
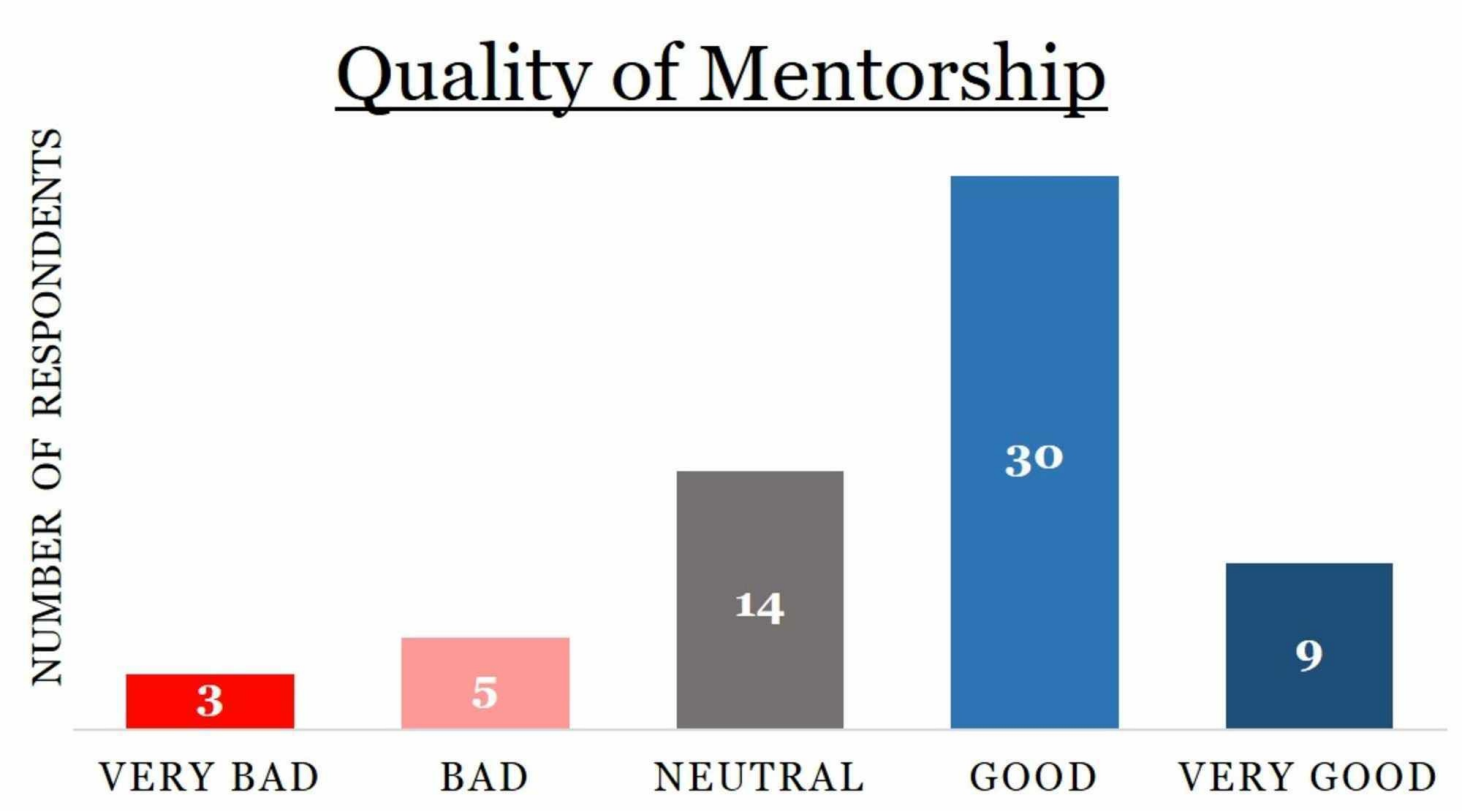
Distribution of survey respondents based on the quality of mentorship they received, with the number of respondents listed within the chart

Regarding the quality of mentorship, 63.9% of respondents reported receiving good (n=30) or very good (n=9) quality of mentorship (Figure 2). Approximately 13% percent of respondents stated the quality of mentorship they received was bad (n= 5) or very bad (n=3) and 23% of respondents (n=14) were neutral in describing the quality of mentorship they received (Figure 2).

**Figure 3:**
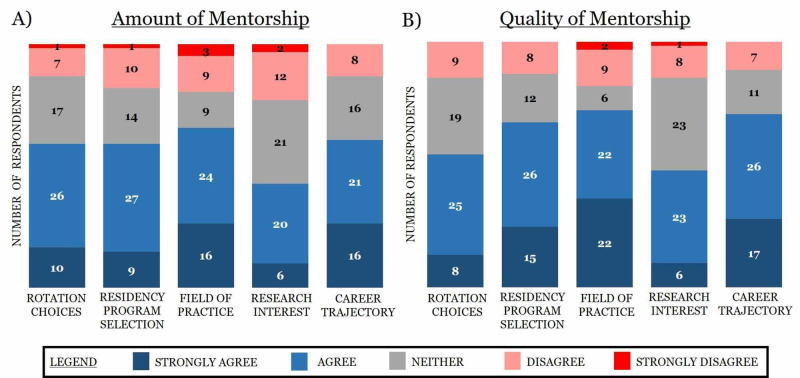
Visual depiction of how the amount and quality of mentorship impact decisions involving rotation choices, residency programs, field of practice, interest in research, and career trajectory Survey respondents were provided five qualitive response answers: strongly agree, agree, neither, disagree, and strongly disagree.

Multiple survey items utilized a conventional ordinal Likert scale (strongly agree, agree, neither, disagree, and strongly disagree) to gauge perceptions. Multiple trends emerged when differentiating participants’ responses to these items. First, most survey respondents strongly agreed or agreed that the amount and quality of mentorship impacted their decisions involving rotation choices, residency program selection, field of practice, and career trajectory (Figure 3). Additionally, interest in research was the only category not to receive a majority of positive responses, which yielded the highest proportion of respondents indicating neutrality (Figure 3). Second, field of practice saw the highest cumulative positive responses (strongly agree and agree) and the least amount of neutral responses. Third, quality of mentorship was perceived as more important than the amount of mentorship in regards to residency program selection and career trajectory, with approximately 10% more positive responses for quality than amount of mentorship (Figure 3).

Separately, 67.2% (n=41) of respondents completed the open-ended narrative question. Qualitative analysis of responses to this item yielded three themes that identified differences in primary care versus specialty medicine, gender differences, and formal versus informal mentorship. Each theme is described and reviewed below, in turn. Each respondent’s unique anonymous identifier listed in the parentheses follows the description.

Primary care versus non-primary care

Narrative responses revealed a marked difference in the perceptions of mentoring based on field of practice. When responses were grouped based on whether participants within the narrative mentioned an interest in primary care or specialty medicine, the majority of students alluding to primary care were positive in their responses and highlighted the mentorship opportunities available in primary care. Interestingly, the majority of students alluding to non-primary care practice were negative in their responses and highlighted the dearth of mentorship opportunities available for them at the institution.

Respondents commented on the differences in the mentorship opportunities available for individuals pursuing a specialty in primary care versus a non-primary care specialty:

“[This institution] really only offers mentoring services to those pursuing primary care. As someone not going into primary care, there were no services available” (Respondent 2).

“I wish that there was more mentorship in specialty medicine” (Respondent 22).

“I even felt a little discouraged at times by faculty members pursuing a field outside of primary care” (Respondent 16).

Additionally, those pursuing a non-primary care specialty felt compelled to seek mentorship outside of their institution:

“As someone entering a specialty [non-primary care] field I had to look outside of my own school for mentorship as most mentorship available at [this institution] is for primary care fields” (Respondent 4).

“I met my mentor at a conference for my intended specialty” (Respondent 24).

These differences in mentorship opportunities suggest a barrier for students pursuing non-primary care specialties and how mentorship could have provided much-needed perspectives on the desired field of practice for a student:

“The college was dedicated to producing Primary Care physicians during the time of my education, I feel that I was left without several resources as I was not entering a Primary Care field. Due to this, I spent a great deal of time agonizing over career choices that could have been much easier with appropriate mentorship” (Respondent 14).

When mentorship opportunities were available for students pursuing a primary care specialty, students took advantage of this mentorship:

“I think mentorship greatly influenced my future career. I was attracted to family medicine because of the great mentors and role models I saw there” (Respondent 12).

“I was strongly impacted by mentorship of multiple primary care sports medicine physicians and that is the career path that I am hoping to follow” (Respondent 18).

Gender differences

**Figure 4:**
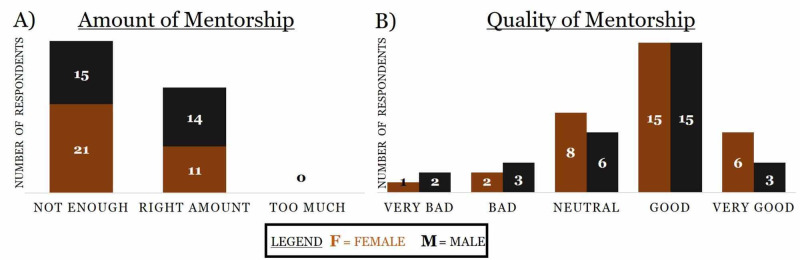
Distribution of respondents based on gender difference and the amount and quality of mentorship they received, with the number of respondents listed within the chart

Quantitative differences were seen in gender-based cohorts. A higher percentage of women (65.6% vs 51.7%) stated that the amount of mentorship they received was not enough (Figure 4A). However, more women (n=21) compared to men (n=18) positively (good and very good) rated the quality of mentorship they received (Figure 4B).

**Figure 5:**
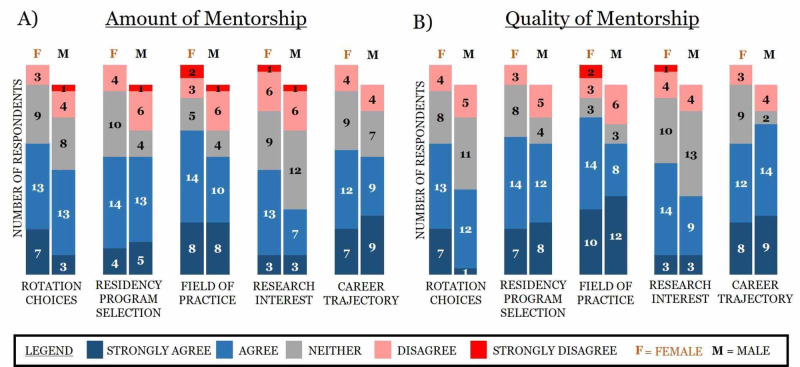
Visual depiction of gender differences based on how the amount and quality of mentorship impact rotation choices, residency programs, field of practice, interest in research, and career trajectory Survey respondents were provided five qualitative response answers: strongly agree, agree, neither, disagree, and strongly disagree.

When gender-based cohorts are stratified by the amount or quality of mentorship they received and if it impacted their decisions involving rotation choices, residency programs, field of practice, interest in research, and career trajectory based on the five ordinal response categories (i.e. strongly agree > strongly disagree), several trends/themes arose. First, approximately 10% more women compared to men indicated that the amount and quality of mentorship positively influenced their interest in research (Figure 5). Second, out of all the categories, more men compared to women indicated that the quality of mentorship positively influenced their career trajectory, the only category in which more men than women were positively influenced by mentorship (Figure 5). Third, approximately 10% more women relative to men indicated that the quality of mentorship positively influenced their decision involving rotation choices (Figure 5).

When the open-ended responses were analyzed by gender differences, specific descriptions from female participants highlighted the shortage of available female mentors:

“I wish that there was more mentorship in specialty medicine. I felt like I was on my own to find mentors (especially female surgery mentors) once I decided that I was not going to pursue primary care” (Respondent 22).

Additionally, female respondents mentioned how mentors exposed them to research:

“I was involved in a summer externship experience where I was exposed to both research and the clinical side of surgery. During this, I was inspired simply by seeing a female trauma surgeon who was extremely successful in the medical field” (Respondent 54).

“I did go to my advisor occasionally who helped me with my interests in research” (Respondent 8).

The impact on rotation choices by mentors was also noted:

“Mentorship impacted where I did audition rotations, applied to residency, and influenced my rank list" (Respondent 22).

“A couple of the surgeons I met during this experience were integral in my choice of specialty and also helped me get a residency position by writing letters of recommendation for me” (Respondent 54).

“Guidance on which rotations to do” (Respondent 59).

Formal versus informal mentorship

When responses were categorized based on formal versus informal mentorship, the majority of students alluded to gaining mentorship from informal mentors with whom they had organically created relationships. These relationships could also be in the form of relatives:

“I relied more on my direct experiences on my rotations and talking to family members who are physicians because I did not feel like we had mentors in medical school” (Respondent 43).

“Having a relative in the field that I matched into had a relatively significant impact on my career choice because not only was I able to ask questions, but I also saw first hand the lifestyle of the profession and what it takes to be successful in that field” (Respondent 36).

Another manifestation of informal mentorship was seen in the form of peer-to-peer mentoring:

“I really had to rely on my friends in residency as my main mentors” (Respondent 58).

“My class figured out most problems by ourselves” (Respondent 23).

Numerous students took the onus on themselves and found mentors in different venues such as professional conferences or other institutions. Many of these students were pursuing a non-primary care specialty:

“As someone entering a specialty [non-primary care] field I had to look outside of my own school for mentorship as most mentorship available at [this institution] is for primary care fields” (Respondent 4).

“My mentorship was from outside of [this institution]” (Respondent 16).

“I will say that this mentor was sought out by myself at a conference and not through [this institution]. Mentorship through [this institution] is difficult to find in some specialties. I feel [this institution] didn’t help at all with mentorship” (Respondent 37).

The scarcity of formalized pathways to develop proper mentorship at the studied institution was identified as an area of improvement:

“I feel [this institution] does not provide enough advising during the matching process. I would have appreciated having a program director that would sit down and give me advice on my field of specialty” (Respondent 58).

“Ultimately, established networking opportunities through mentorship opened doors for interviews and possibilities for career trajectory [versus an] attending who would invest little to no effort in the student” (Respondent 44).

Formalized pathways of mentorship can supplement the informalized pathways of finding a mentor and build a relationship organically. In one instance, a medical student mentioned a mentor-mentee relationship formally forged yielding positive results:

“[This institution] put in contact with a graduate in my specialty. She greatly assisted me in getting into the program that I matched in” (Respondent 53).

## Discussion

The respondents’ quantitative and qualitative responses corroborate our hypotheses. The respondents indicated their mentorship experiences had a strong impact on their decisions involving rotation choices, residency programs, field of practice, and career trajectory. However, medical students at this institution believed the amount and quality of mentorship could be improved. This perception is not limited to this institution, as in a separate study, international medical students believed opportunities for mentorship were not sufficiently available [9]. In addition, close to half of the medical schools in Germany did not offer mentoring programs to students according to a previous study investigating the mentorship of German medical students [9]. The perceived lack in the amount and quality of mentorship in our sample may have profound negative implications on medical students’ career prospects because mentorship provides guidance in the residency application process, assists students in making thoughtful decisions on their career, enhances professionalism in mentees, improves medical school performance, and enhances interest in research, among other positive outcomes [4]. Without the appropriate availability of mentorship, medical students eager for this resource may be at a disadvantage in terms of preparation for residency applications, professional development, and discovering an interest in an academic career. Literature has demonstrated an improvement in clerkship grades, increased match rate, and enhanced residency applications for specialties that provided mentorship to medical students [10].

The lack of mentorship opportunities cannot be generalized amongst all demographics. As compared to males, females in our study were less satisfied with the number of mentorship opportunities available to them. This is even more detrimental as a significantly higher proportion of females compared to male students have been found to value mentorship as a key contributor to personal development [11]. It is important to focus on these gender differences now, particularly since females recently became a majority, for the first time ever, of United States medical students [12]. Moreover, female academic health professionals identified mentorship availability as key to their success [11]. Additionally, in our study, more female respondents indicated that mentorship had a positive influence on their research interests. A separate study found that male respondents were more interested in a career in academic medicine as compared to female respondents, a career in which there is an impetus on research productivity [8]. A lack of mentorship opportunities may preclude females from pursuing academic medicine and may be one possible explanation for gender disparities in academic medicine [13].

Interestingly, more male respondents indicated that the quality of their mentorship experiences had a positive influence on career trajectory as compared to female respondents. This may be a barrier for female respondents when evaluating career trajectory. Previous research has indicated that female mentees may require a supportive and collaborative mentoring model and, as a result, new mentoring models have emerged in medicine [14]. It appears one type of mentoring model may not be as effective for both men and women uniformly and that multiple mentoring approaches should be available and tailored to the individual.

The results of this study inform the important role of mentorship in a medical student’s career intentions. As previously stated, students intending to pursue non-primary care specialties had a lack of both formal and informal mentorship opportunities at this institution, putting the burden on them to seek proper guidance to pursue their specialty of choice. Although other schools might not have this particular focus on primary care, it is important that all medical educators be mindful of potential biases for one group or another. Proper mentorship has been found to aid students in research opportunities, career advice, and guidance in the residency application process [4]. Among emergency medicine residency applicants, matching into that specialty has been correlated with the degree of mentorship a student has received [5]. Students in our study who were interested in a non-primary care specialty could potentially have had their residency prospects hindered due to the lack of mentorship opportunities available for non-primary care specialties.

Our results corroborate previous studies in which respondents utilized both formal and informal mentorship. However, in our study, the lack of formal mentorship was identified as an area of improvement at this institution. A systematic review of mentorship did not conclusively identify one form of mentorship to be more effective than the other [2]. Based on our results, it is imperative that medical students have access to both styles of mentorships. Formalized mentorship may provide medical students with the network to seek out informal, organic mentorship. Additionally, for individuals who are unable to build constructive and positive mentorship through informal mechanisms, formalized mentorship may serve as a safety net for them so they can receive adequate mentorship.

Interestingly, our results also found that no medical student indicated receiving excessive mentorship, suggesting that excessive mentorship may not possibly exist. The amount of mentorship an individual requires is variable, but mentees may pick and choose which advice to heed, thereby not perceiving an excess of mentorship. Consequently, excessive mentorship may provide more viewpoints that may provide the mentee with a more complete understanding of a situation. However, one may consider how much mentorship can be provided to a mentee in helping them reach their potential. Although mentor availability has been identified as a barrier to mentorship, further studies can investigate the amount of time spent with a mentor to yield the most benefits to the mentee to determine the optimal amount of mentorship [15].

Admittedly, more research is needed to further reveal the impact of mentorship on medical students. Future considerations include, but are not limited to, allopathic medical student mentorship experiences, how it compares to the mentorship experiences of osteopathic medical students, and how mentorship experiences change in residency once a medical trainee enters their field of choice. Furthermore, additional research is needed to understand the impact of poor mentorship on medical students’ decision-making regarding rotation choices, residency program choices, field of practice, interest in research, and career trajectory.

## Conclusions

This study demonstrates the important role of mentorship in a medical student's journey in medical school. One overall conclusion is the perceived scarcity of mentorship opportunities, especially among those pursuing non-primary care specialties. This may have negative consequences for students who seek to match into a competitive specialty. Additionally, gender differences were identified, as female respondents indicated a lack of mentorship opportunities. Regardless, the principles of mentorship can influence the continuous quality improvement of medical education at other institutions such as improving mentorship availability and creating formal pathways to supplement informal mentorship. Mentorship’s vital role in the development of medical students is not well understood, however, its implications for the professional development of physicians are profound.

## Appendices

Survey

Q1

You are being asked by an Ohio University researcher to participate in research. For you to be able to decide whether you want to participate in this project, you should understand what the project is about, as well as the possible risks and benefits in order to make an informed decision. This process is known as informed consent. This form describes the purpose, procedures, possible benefits, and risks of the research project. It also explains how your personal information will be used and protected. Once you have read this form and your questions about the study are answered, you will be asked to participate in this study. You may print a copy of this document to take with you. Summary of Study

This study aims to understand the role mentorship plays in medical education and decisions made by medical students regarding their career. Based on prior research, mentorship has been found to affect career transitions in medicine, but never specifically mentioning medical student’s transition to residency. Prior research has also found that race, gender, and minority-status impact differences in mentorship. Our goal is to understand mentorship's impact on perceived rotation choices, residency programs, field of practice of medicine, interest in research, and career trajectory. We also seek to understand if an individual who has a family member in the field of medicine is more likely to receive mentorship. We hope to assess mentorship by the amount received and the quality of mentorship.

This study will be conducted via an online anonymous survey. Explanation of Study

This study is being done to understand the role mentorship plays in medical education and decisions made by medical students regarding their career. If you agree to participate in this online anonymous survey, you will be asked to complete a 9 question survey that asks your race/ethnicity, your gender, if you have a relative who is a physician, the amount and quality of mentorship received. Additionally, you will be asked to evaluate statements at the end of the survey and briefly explain how mentorship in medical school impacted your future career. You should not participate in this study if you are not a fourth year medical student. Your participation in the study will require 5 to 7 minutes to complete the survey. Risks and Discomforts

No risks or discomforts are anticipated Benefits

This study is important to science/society because it will help us understand the role mentorship plays in medical education and decisions made by medical students regarding their career. This study helps to inform future medical education initiatives that incorporate mentorship. Confidentiality and Records

Your study information will be kept confidential by the survey refraining from asking your name. For maximum confidentiality, please clear your browser history and close the browser before leaving the computer. Compensation

None. Future Use Statement

Identifiers might be removed from data/samples collected, and after such removal, the data/samples may be used for future research studies or distributed to another investigator for future research studies without additional informed consent from you or your legally authorized representative. By agreeing to participate in this study, you are agreeing that: ·         you have read this consent form (or it has been read to you) and have been given the opportunity to ask questions and have them answered; ·         you have been informed of potential risks and they have been explained to your satisfaction; ·         you understand Ohio University has no funds set aside for any injuries you might receive as a result of participating in this study; ·         you are 18 years of age or older; ·         your participation in this research is completely voluntary; you may leave the study at any time; if you decide to stop participating in the study, there will be no penalty to you and you will not lose any benefits to which you are otherwise entitled. By clicking "Yes" below, you will consent to participate in this short survey. Do you consent to participate in this study? •     Yes, I consent (I may withdraw participation at any time). •     No, I do not consent.

Q2

With which of the following do you primarily identify? •     Male •     Female •     Other

Q3

With which of the following do you primarily identify? •     White •     Black or African American •     American Indian or Alaska Native •     Asian •     Native Hawaiian or Pacific Islander •     Other

Q4

Do you have a relative that is a physician? A "relative" is defined as any of the following: (parent, grandparent, sibling, aunt, uncle, or cousin). •     Yes •     No

Q5

How much Mentorship did you receive in Medical School? •     Not Enough •     The Right Amount •     Too Much

Q6

What was the quality of Mentorship you received in Medical School? •     Very Bad •     Bad •     Neutral •     Good •     Very Good

Q7

The amount of mentorship I received during Medical School (regardless of how much or how little) has affected... Strongly Agree    Agree    Neither    Disagree    Strongly Disagree ...my rotation choices ...my residency program selection ...my field of practice ...my interest in research ...my career trajectory Q8

The quality of mentorship I received during Medical School (regardless of how much or how little) has affected... Strongly Agree    Agree    Neither    Disagree    Strongly Disagree ...my rotation choices ...my residency program selection ...my field of practice ...my interest in research ...my career trajectory Q9

Regardless of amount or quality of mentorship you received, briefly explain below how mentorship in medical school impacted your future career.
